# Long-term effects of climate factors on dengue fever over a 40-year period

**DOI:** 10.1186/s12889-024-18869-0

**Published:** 2024-05-30

**Authors:** Chengdong Xu, Jingyi Xu, Li Wang

**Affiliations:** 1 State Key Laboratory of Resources and Environmental Information System, Institute of Geographical Sciences and Natural Resources Research, Chinese Academy of Sciences, Beijing, China; 2 College of Resources and Environment, University of Chinese Academy of Sciences, Beijing, China; 3 College of Geography and Environmental Science, Henan University, Kaifeng, China; 4 Key Laboratory of Geospatial Technology for the Middle and Lower Yellow River Regions (Henan University), Ministry of Education, Kaifeng, China

**Keywords:** Dengue, Long-term, Climate

## Abstract

**Background:**

Dengue fever stands as one of the most extensively disseminated mosquito-borne infectious diseases worldwide. While numerous studies have investigated its influencing factors, a gap remains in long-term analysis, impeding the identification of temporal patterns, periodicity in transmission, and the development of effective prevention and control strategies. Thus, we aim to analyze the periodicity of dengue fever incidence and explore the association between various climate factors and the disease over an extended time series.

**Methods:**

By utilizing monthly dengue fever cases and climate data spanning four decades (1978–2018) in Guangdong province, China, we employed wavelet analysis to detect dengue fever periodicity and analyze the time-lag relationship with climate factors. Additionally, Geodetector q statistic was employed to quantify the explanatory power of each climate factor and assess interaction effects.

**Results:**

Our findings revealed a prolonged transmission period of dengue fever over the 40-year period, transitioning from August to November in the 1970s to nearly year-round in the 2010s. Moreover, we observed lags of 1.5, 3.5, and 3 months between dengue fever and temperature, relative humidity, and precipitation, respectively. The explanatory power of precipitation, temperature, relative humidity, and the Oceanic Niño Index (ONI) on dengue fever was determined to be 18.19%, 12.04%, 11.37%, and 5.17%, respectively. Dengue fever exhibited susceptibility to various climate factors, with notable nonlinear enhancement arising from the interaction of any two variables. Notably, the interaction between precipitation and humidity yielded the most significant effect, accounting for an explanatory power of 75.32%.

**Conclusions:**

Consequently, future prevention and control strategies for dengue fever should take into account these climate changes and formulate corresponding measures accordingly. In regions experiencing the onset of high temperatures, humidity, and precipitation, it is imperative to initiate mosquito prevention and control measures within a specific window period of 1.5 months.

## Background

Currently, vector-borne diseases pose a threat to over 80% of the world's population, with mosquito-borne illnesses contributing the most to the overall disease burden [1]. The most prevalent viral infection transmitted by Aedes mosquitoes, dengue fever affects at least 128 countries and is thought to cause between 50 and 100 million cases annually [2, 3]. Aedes mosquitos help spread dengue disease to humans because they are highly adapted to urban settings in tropical and subtropical regions of the world [4].

A variety of factors influence dengue fever incidence and transmission. Extensive studies have established the importance of climate parameters such as temperature, precipitation, and humidity in driving dengue fever dynamics [1, 5, 6]. The dynamics of mosquito growth, the spread of viruses, and mosquito-human interactions are all influenced by the climate [7]. Temperature plays a crucial role in vector development, biting rates, and the rate of pathogen development within mosquitoes [8]. Since stagnant water pools are necessary for mosquito breeding and growth, precipitation is an important element in climate models used to identify dengue fever. The chance of dengue transmission can be impacted by humidity, which can also have an impact on mosquito survival and flight patterns [1, 9].

In addition to these commonly studied climate factors, extreme weather occurrences can also significantly affect the incidence of dengue disease [10, 11]. The El Niño-Southern Oscillation (ENSO), which has a periodicity of two to seven years and is defined by alternating warm and cool phases in the tropical Pacific, is a persistent and irregular phenomena. Anomalies in the world's temperature and precipitation patterns are brought on by ENSO events. According to several studies, ENSO significantly affects dengue fever dynamics by accounting for about 40% of the fluctuation in temperature and rainfall [11].

Southern and eastern China are included in the East Asian monsoon region, which is highly sensitive to climate change. The dengue fever epidemic, which has had a considerable influence on these areas over the past few decades and is defined by different temporal and spatial evolution patterns, has had a significant impact on these areas. There were no known dengue fever cases in China until 1977. However, a dengue epidemic in the province of Guangdong in 1978 heralded the start of the illness' intermittent predominance across the country [12]. Then, dengue fever outbreaks were reported in Hainan, Guangxi, Fujian, and Zhejiang provinces sequentially [12]. In mainland China, a total of 655,324 cases and 610 fatalities were recorded from 1978 to 2008, and 52,749 cases and 6 fatalities from 2009 to 2014 [13]. As a result, dengue fever has grown to be a serious illness in China and was classified as an infectious disease of type B in 2004. Despite the fact that there have recently been a few dengue fever cases in inland regions like Henan province [14], 94.3% total of dengue cases in mainland China were from Guangdong province [15], where the hot, humid weather conditions are favorable for mosquito breeding and dengue disease transmission [16]. Especially, in 2014 China has undergone the worst dengue outbreak in the last 20 years [17], which caused great concern about dengue fever in China.

Many studies have focused on the temporal dynamics of dengue fever incidence over a period of 10 to 25 years [6, 18–20]. However, there is limited research on the epidemic patterns of dengue fever and the effect of climate factors on dengue fever under long-term climate change (climate change refers to climate variations typically spanning 30 years or longer). Between the mid-twentieth century and 2018, climate change increased the probability of transmission by 15.0% for Aedes albopictus and 8.9% for Aedes aegypti, the principal vectors of dengue [21]. As global warming continues, several locations, including China's eastern coast, are expected to become appropriate breeding grounds for the dengue virus by 2050 [22]. Hence, it is important to conduct a comprehensive analysis of dengue dynamics over a prolonged historical period to understand the relationship between climate and dengue for formulating effective strategies for early warning, prevention, control of dengue fever, and informing public health measures aimed at reducing the burden of dengue fever and guide future research efforts in the field of vector-borne diseases.

This study aimed to analyze the periodicity of dengue fever incidence, evaluate the lag relationship between climate factors and the disease, and assess the explanatory power and interaction effects of these climate variables using dengue fever and climate data from Guangdong province spanning the years 1978 to 2018.

## Methods

### Data

Our study incorporated three distinct datasets. Firstly, we utilized monthly dengue fever cases in Guangdong province spanning the years 1978 to 2018. These data were sourced from previous papers [23–26]. Secondly, we obtained meteorological data, including monthly mean temperature, monthly mean relative humidity, and monthly total precipitation, from the China Meteorological Data Sharing Service System (http://data.cma.cn/). Lastly, we incorporated the monthly Oceanic Niño Index (ONI) as an indicator for the El Niño-Southern Oscillation (ENSO) phenomenon. The ONI data, representing the sea surface temperature anomaly index for the Niño region 3.4, were acquired from the Climate Prediction Center of the National Weather Service (https://origin.cpc.ncep.noaa.gov). To supplement our analysis, we accessed the monthly population data of Guangdong province from the Guangdong Statistics Yearbook. The multicollinearity test showed that all climate factors had VIF values less than 3, so there the possibility of multicollinearity was ruled out.

### Wavelet analysis

When analyzing time series that contain non-stationary power at various frequencies, the wavelet transform is always applied. A wavelet is a zero-mean function that is confined in frequency and temporal space. There are many wavelets that have been characterized, including the Morlet, Paul, and Gaussian derivative, each of which has appropriate application conditions [27]. In our study, we chose the Morlet wavelet ($\omega_0$ = 6), which is considered to provide a good balance between time and frequency localization [28]. The Morlet is defined as:

1 $$\psi_0(\eta)=\pi^{-1/4}e^{{iw}_0\eta}e^{-\eta^2/2}$$

where $w_0$ and $\eta$ are dimensionless frequency and time, respectively. The wavelet is stretched in time by changing its scale (s) to $\eta=s\times t$, and normalized to have unit energy. The wavelet power is defined as $\left|W_n^X(s)\right|^2$. The specific formula is as follows:

2 $$W_n^X(s)=\sqrt{\frac{\delta_t}s}\sum\limits_{n'=1}^Nx_{n^{'}}\psi_0\left[(n^{'}-n)\frac{\delta_t}s\right]$$

where $W_n^X(s)$ is the transformed time series for scale *s*; $\delta_t$ is the time interval; $n$ is the time and $n'$ is the reversed time. Then a wavelet power spectrum is generated to explore the periodicity of each time series. The time series were padded with enough zeros to generate a time series of length since working with finite-length time series will result in inaccuracies at the start and end of the wavelet power spectrum. The cone of influence (COI) is used to depict regions that give erroneous results in order to remove the impact of discontinuities at the endpoints and the lowering of edge amplitude influenced by zero-padding [27].

Cross Wavelet Transform (XWT) is used to probe coincident high power between two time series which is defined as:

3 $$W^{XY}=W^XW^{Y^\ast}$$

where * means complex conjugation. The XWT power is defined as $\left|W^{XY}\right|$. There is also a power spectrum in XWT, where the arrows represent the relative phase [28]. Phase arrows pointing right mean the two variables are in phase, and the left arrows mean anti-phase. Down arrows mean X leads Y by 90 degrees, and up arrows mean Y leads X by 90 degrees [29].

### Geodetector* q* statistic

In order to explore the association between long-term climate factors and the incidence of dengue fever, and reveal the interaction between these climate factors, Geodetector *q* statistic was applied. The basic principle of the method is to assume that if the spatial or temporal distribution of two variables tends to be consistent, there is statistical association between them [30]. Our study aimed to explore the impact of different factors on the variation in time of dengue fever incidence.

In our study, *q* statistic of Geodetector were used to illustrate the explanatory power of independent variables:

4 $$q=1-\frac{{\sum\limits_{h=1}^L}N_h\sigma_h^2}{{N\sigma}^2}=1-\frac{SSW}{SST}$$

where* q* is the explanatory power of each factor on dengue fever incidence, which follows a Noncentral-F distribution [30, 31] and the range is from 0 to 1. The *q* value indicates that X explains 100 × q% of Y, the larger the value is, the stronger the explanatory power of independent variable X to Y is, and vice versa. *h* is the number of the strata of variable X (climate factors); $N_h$ and N are the sample size in the *h-*th stratum and the whole regions, respectively; $\sigma_h^2$ and $\sigma^2$ are the variance of Y (dengue fever incidence) for the *h-*th stratum and the whole regions; SSW and SST indicate the within sum of squares and total sum of squares, respectively.

Interaction detector of Geodetector can be used to assess the interaction effect of two variables, e.g.,*X*_*1*_ and *X*_*2*_, which can calculate the explanatory power *q(X*_*1*_* ∩ X*_*2*_*)* of two factors and probes whether the explanatory power of two factors is enhanced or weakened when taken together, or whether they are independent by comparing *q(X*_*1*_* ∩ X*_*2*_*)* with *q(X*_*1*_*)* and *q(X*_*2*_*)*. The description of each interaction demonstrates in Table 1 [32, 33].

**Table 1:** The interaction relationship of Geodetector *q* statistic

Interaction relationship	Interaction effect
$q (X_1\cap X_2)$ < $Min(q(X_1),\;q(X_2))$	Weaken, nonlinear
$Min(q(X_1),\;q(X_2))$ < $q(X_1\cap X_2)$ < $Max(q(X_1),\;q(X_2))$	Weaken, univariate
$q(X_1\cap X_2)$ > $Max(q(X_1),\;q(X_2))$	Enhance, bivariate
$q(X_1\cap X_2)$ = $q(X_1)$ + $q(X_2)$	Independent
$q(X_1\cap X_2)$ > $q(X_1)$ + $q(X_2)$	Enhance, nonlinear

$X_1\cap{X_2}$ means the new stratum created by overlaying *X*_1_ and *X*_2_

## Results

### Periodicity of dengue fever and climate factors

**Fig. 1:**
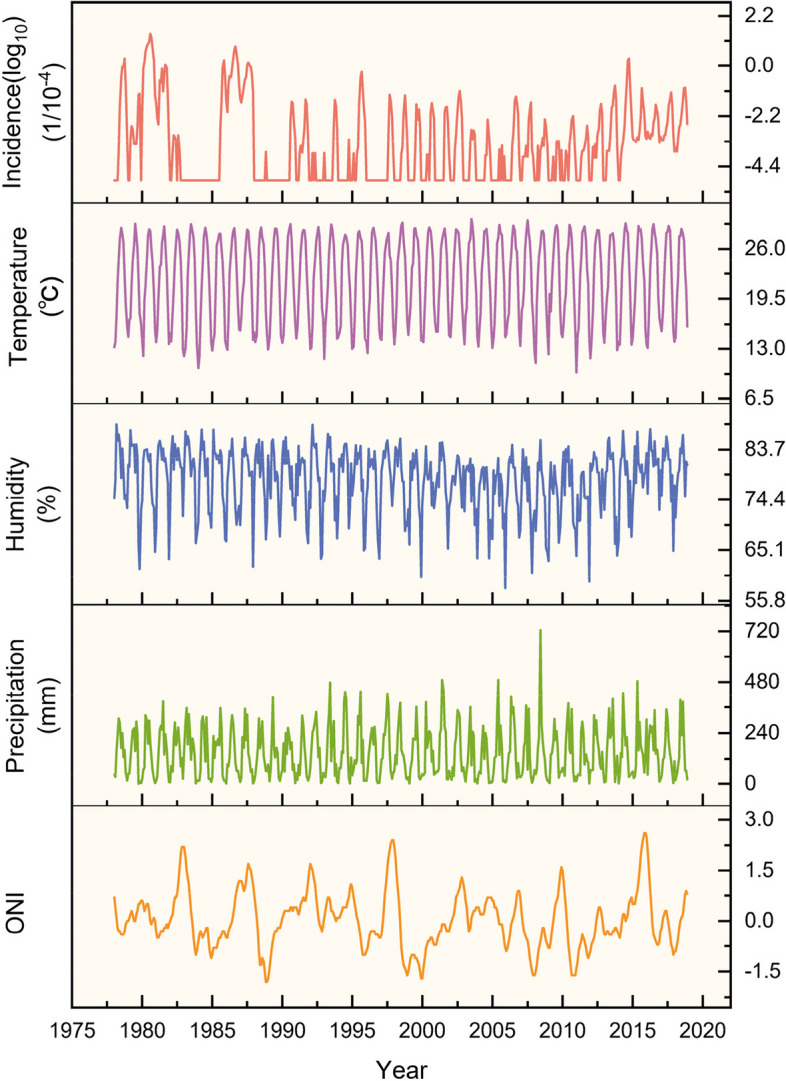
Temporal dynamics of dengue fever incidence and associated variables, 1978–2018. From top to bottom, the figure shows monthly dengue fever incidence (log_10_), average temperature, relative humidity, precipitation and ONI

The 40-year time series analyzed in this study revealed noticeable fluctuations in dengue fever incidence. Specifically, there were seven years during which the number of dengue fever cases exceeded 10,000: 452,674 in 1980, 118,881 in 1986, 45,189 in 2014, 32,830 in 1987, 22,122 in 1978, 19,543 in 1981, and 16,385 in 1985 (Fig. 1). In comparison to these high-incidence years, the occurrence of dengue fever in other years was relatively lower.

**Fig. 2:**
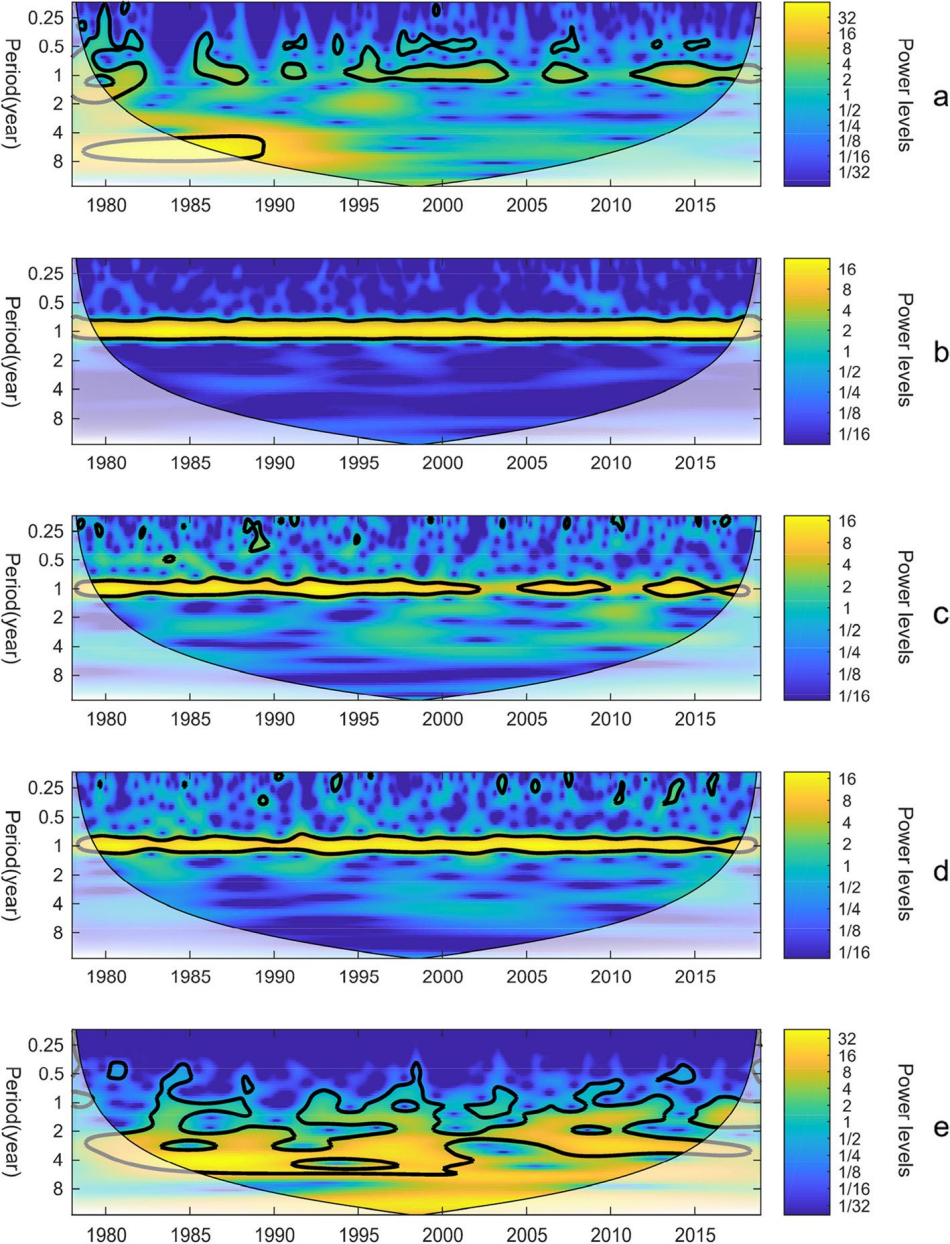
Continuous wavelet transform power spectrum of dengue fever incidence and climatic factors, 1978–2018. **a**-**e** dengue fever incidence (**a**) average temperature (**b**) average relative humidity (**c**) precipitation (**d**) EI Niño index (**e**). The thick black contour designates the 5% significance level against red noise and the cone of influence (COI) where edge effects might distort the picture is shown as a lighter shade

To assess the periodicity of dengue fever incidence and its association with climate factors, we conducted Continuous Wavelet Transform (CWT) analysis on these variables (Fig. 2). The wavelet power spectrum revealed distinct scales of periodicity in dengue fever incidence during specific time intervals, namely 1980–1982, 1985–1987, 1994–2004, 2006–2007, and 2011–2016, dengue fever incidence was composed of different scales periods: a distinct one-year cycle and a cycle of half a year. Notably, over the 40-year period, the prevalence of the half-year cycle gradually diminished, transitioning into a one-year cycle. This observation implies an extended transmission period for dengue fever, encompassing nearly the entire year instead of being limited to August to November. Additionally, a five-year cycle was evident during the 1980s, indicating notable inter-annual variation in dengue fever incidence (Fig. 2a).

The wavelet power spectra analysis revealed a consistent one-year cycle in temperature, relative humidity, and precipitation. This cycle showed regular inter-annual variation throughout the entire time series (Fig. 2b, c, d). Notably, among the analyzed climate factors, the El Niño Index (ONI) exhibited distinct characteristics compared to others. Over the entire time series, the periodicity of the El Niño phenomenon was observed within the range of 2–7 years, with a particular emphasis on the 3–6 year interval, indicating an average occurrence every 3–6 years (Fig. 2e).

### Time-lag association between dengue fever and climate factors

**Fig. 3:**
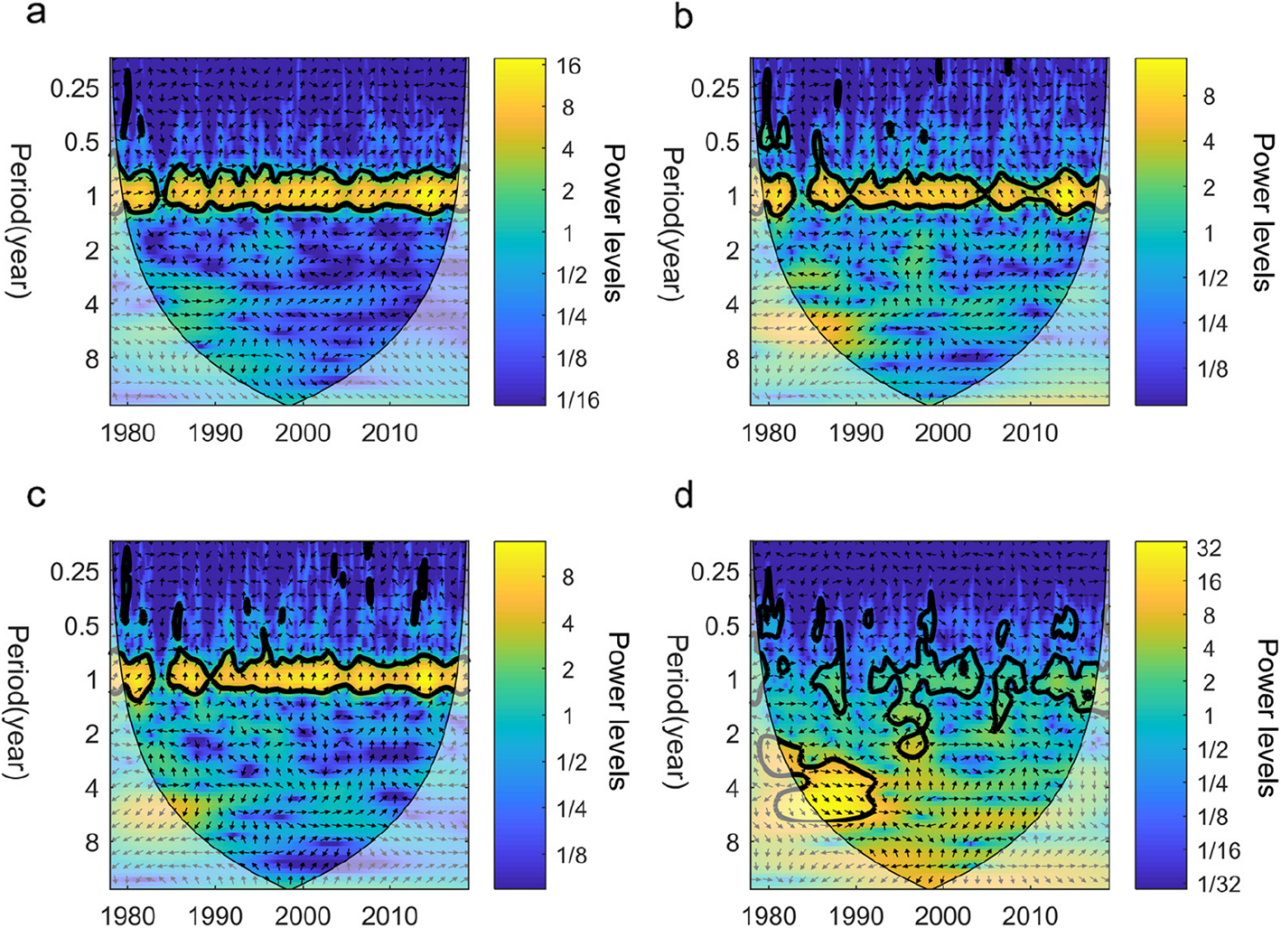
Cross-wavelet power spectrum of dengue fever incidence and climatic factors in Guangdong Province, 1978–2018. **a**-**d** dengue fever incidence—average temperature (**a**) dengue fever incidence—average relative humidity (**b**) dengue fever incidence – precipitation (**c**) dengue fever incidence—EI Niño index (**d**). The thick black contour designates the 5% significance level against red noise and the cone of influence (COI) where edge effects might distort the picture is shown as a lighter shade

From each two CWTs (one is dengue fever incidence and the other is a climate factor), we performed Cross Wavelet Transform (XWT) to assess their shared power and relative phase in the time–frequency domain, thereby investigating the association between dengue fever incidence and climate factors (Fig. 3).

The XWT of dengue fever incidence and temperature shows that they have common high power in one year over the whole time series, so the temperature is a significant factor that influences the dengue fever. The mean phase of the XWT phase angle outside the COI and inside the 5% significant areas is 45 ± 15°. It indicates that within one year, the two factors are in phase and the incidence of dengue fever lags behind the average temperature by 1/8 cycles, implying that the incidence of dengue fever lags behind the average temperature by about 1.5 months (Fig. 3a).

The XWT of dengue fever incidence and relative humidity shows that they also have common high power in one year over the whole time series, and the mean phase of the XWT phase angle outside the COI and inside the 5% significant areas is 105 ± 15°. It means that within one year, the incidence of dengue fever lags behind the relative humidity by 7/24 cycles, implying that the incidence of dengue fever lags behind the relative humidity by about 3.5 months (Fig. 3b).

The XWT of dengue fever incidence and precipitation shows that they are in phase with significant common power in one year during the 40 years. The XWT phase angle has a mean phase of 90 ± 15°, which means that within one year, the incidence of dengue fever lags behind the precipitation by 1/4 cycles, implying that the incidence of dengue fever lags behind the precipitation by about 3 months (Fig. 3c).

The relationship between dengue fever incidence and the El Niño Index (ONI) exhibits a relatively complex nature. The Cross Wavelet Transform (XWT) analysis reveals a significant shared power in the 3–6 year band, particularly during the period from 1984 to 1992, indicating a strong correlation between the two variables. Furthermore, throughout the entire study period, they also exhibit consistent power in the one-year band, further indicating a correlation between these factors on an annual basis (Fig. 3d).

The Cross Wavelet Transform (XWT) revealed the degree of correlation between climate change and dengue fever cases in the time–frequency space, providing knowledge about time lags. To further validate these computational results, we used another method, the Spearman coefficient, to calculate the impact of different lag periods of various climate factors on dengue fever cases, as shown in Table 2. Temperature showed a significant positive correlation with dengue fever incidence at different lag times, with the strongest correlation at a lag of 2 months, with a correlation coefficient of 0.459; this was followed by a lag of 1 month with a correlation coefficient of 0.431. Relative humidity also exhibited a significant positive correlation with dengue fever incidence at different lag times, with the strongest correlation at a lag of 3 months, with a correlation coefficient of 0.333; this was followed by a lag of 4 months with a correlation coefficient of 0.305. Precipitation and the Oceanic Niño Index (ONI) also showed positive correlations with dengue fever incidence at different lag periods. Among them, precipitation exhibited the strongest correlation at lag times of 2 and 3 months, with correlation coefficients of 0.371 and 0.353, respectively. ONI, on the other hand, showed lower correlations with dengue fever incidence in lag periods from 0 to 5 months, indicating longer-term interannual lag effects. These results are consistent with the Cross Wavelet Transform (XWT), demonstrating the reliability of the findings in this study.

**Table 2:** Time-lag analysis results between climate factors and dengue fever incidence

	Lag0	Lag1	Lag2	Lag3	Lag4	Lag5
Temperature	0.280^**^	0.431^**^	0.459^**^	0.368^**^	0.181^**^	-0.049
Humidity	-0.051	0.142^**^	0.280^**^	0.333^**^	0.305^**^	0.239^**^
Precipitation	0.089^*^	0.275^**^	0.371^**^	0.353^**^	0.281^**^	0.113^*^
ONI	0.133^**^	0.119^**^	0.103^*^	0.096^*^	0.090^*^	0.079

^*^p < 0.05, ^**^p < 0.01

### Influence of various climate factors on dengue fever incidence

**Fig. 4:**
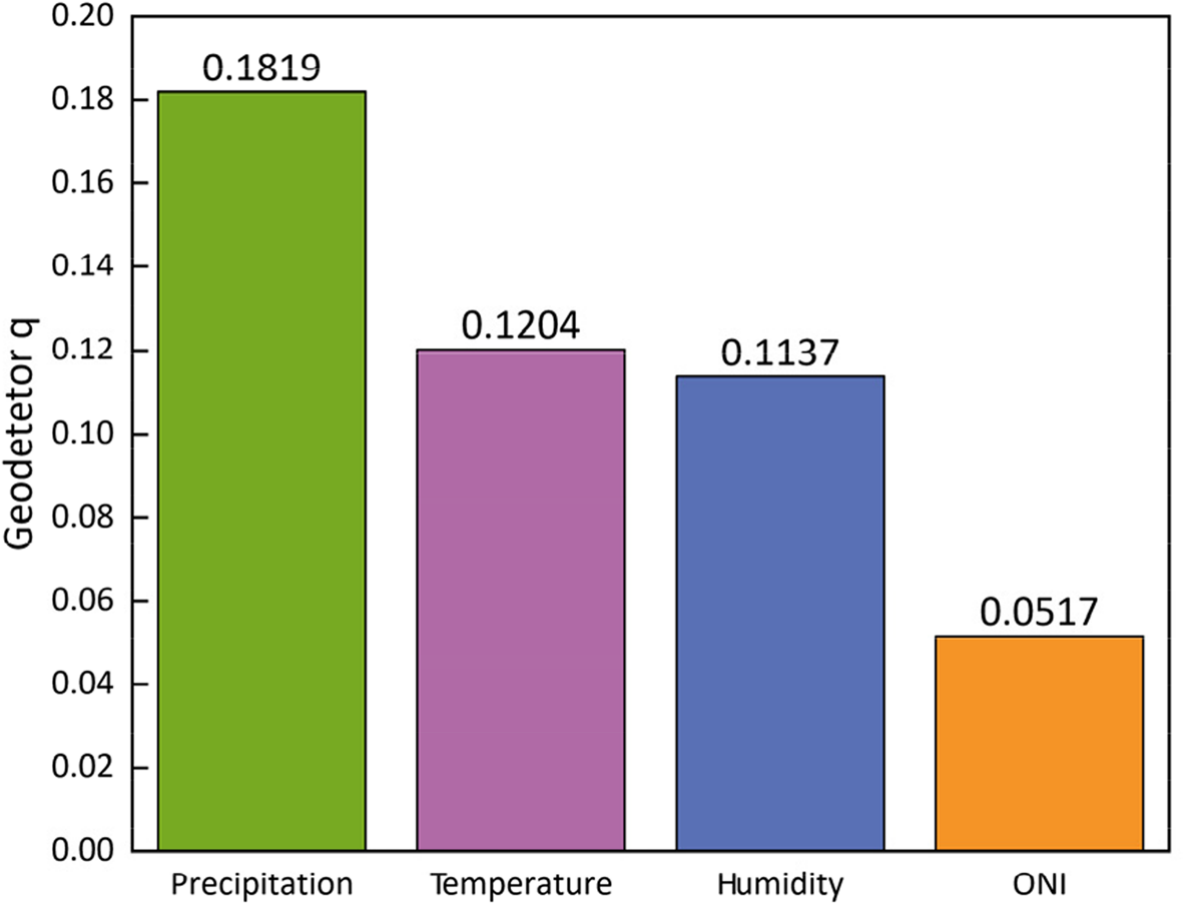
Geodetector q statistic of various climate factors

Based on the XWT analysis, it is evident that temperature, relative humidity, precipitation, and the El Niño Index (ONI) play significant roles in influencing the incidence of dengue fever. To quantitatively assess their explanatory power, we employed Geodetector. The geodetector q statistic revealed that precipitation, temperature, and relative humidity accounted for 18.19%, 12.04%, and 11.37% of the heterogeneity in dengue fever incidence, respectively, while the influence of the El Niño Index was measured at 5.17% (Fig. 4). All the findings were statistically significant.

**Fig. 5:**
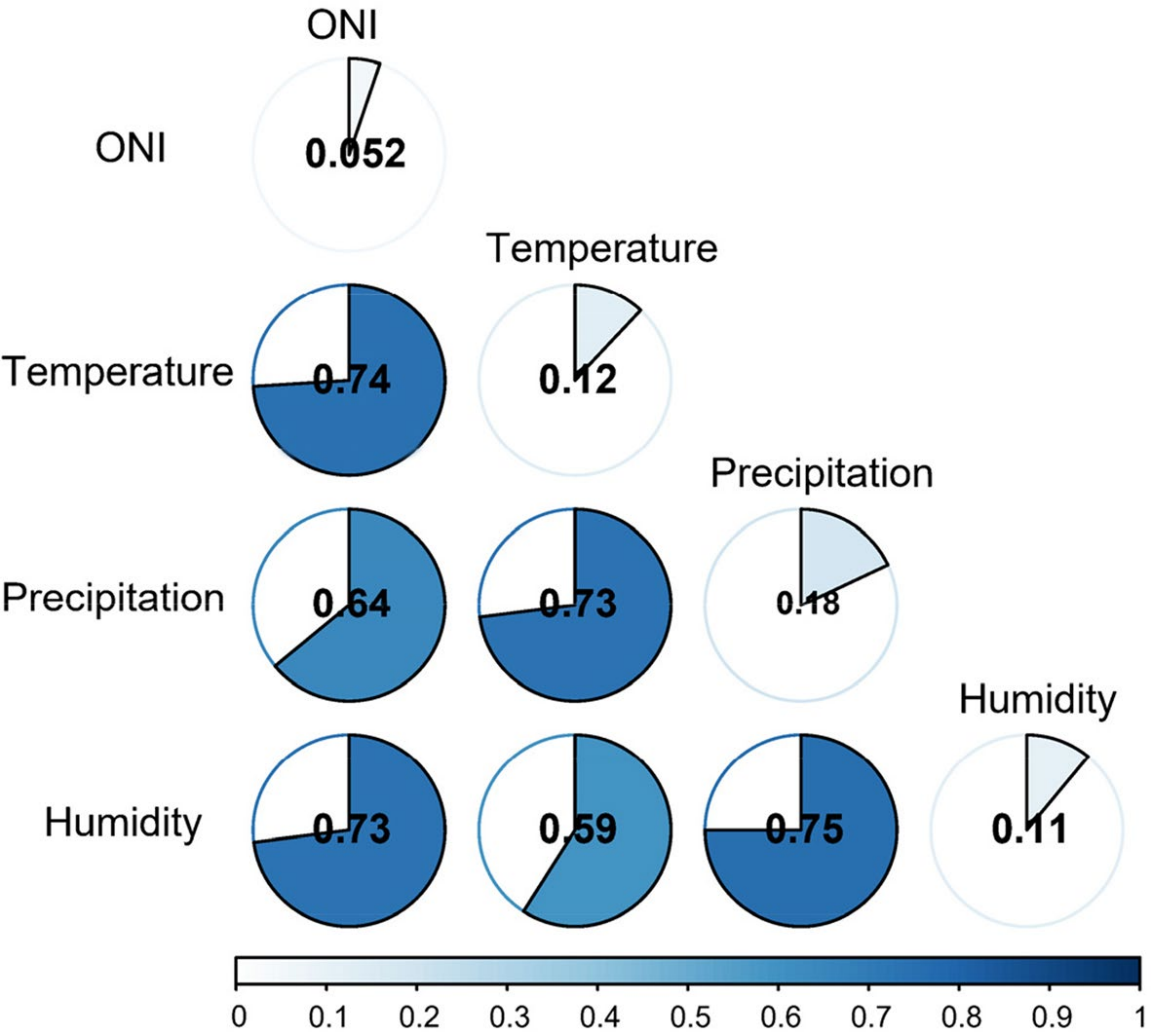
The interaction effect of climate factors on dengue fever

To examine the interactive effect of climate factors on the incidence of dengue fever, we conducted geodetector analysis to calculate the interaction detector for each pair of factors. The results are presented in Fig. 5. Notably, the analysis reveals that the interaction between any two variables leads to a nonlinear enhancement in their influence. Particularly, when considering the combined effect of temperature, precipitation, and relative humidity, their impact on dengue incidence surpasses 70% of the geodetector q statistic. Furthermore, the explanatory power of the El Niño Index (EI Niño) significantly improves when interacting with any other factor (Fig. 5).

## Discussion

Dengue fever is considered to be a disease strongly influenced by climate. Climate change can have both direct and indirect impacts on the ecology of dengue fever. Numerous empirical studies have investigated the relationship between dengue fever and climate factors using various analytical approaches [7, 29, 34, 35]. However, the long-term effects of climate factors on dengue fever remain unclear. In this study, we conducted wavelet analyses on a time series of monthly reported dengue cases and climate variables spanning from 1978 to 2018. The aim was to detect the periodicity of dengue fever and climate factors and qualitatively demonstrate their phase and time-lag relationship. Additionally, we employed Geodetector to quantitatively assess the relative importance of each climate factor on dengue fever and their interactions. Our findings revealed that dengue fever exhibits noticeable inter-annual and intra-annual variations, with different associations observed between the disease and certain climate factors.

Our study uncovered a novel finding regarding the changing periodicity of dengue fever over time. Specifically, we observed a shift from a half-year cycle to a one-year cycle, indicating a lengthening of the epidemic period for dengue fever. This intriguing observation suggests that the influence of global warming and urbanization might contribute to this phenomenon [36]. A periodicity of two to three years has been mentioned in a few earlier studies conducted in Southeast Asian nations like Thailand and Vietnam [37, 38]. Our study suggests that Guangdong has a relatively low inter-annual variation in dengue fever which is stable in both the short and long term. This also supports the conclusion that when the length of the warm season is short, dengue fever cycles in higher latitudes are shorter than those in lower latitudes [20]. Guangdong's lower inter-annual variation is most likely caused by the reason that dengue fever in China is still an imported illness rather than an indigenous one. Although Southeast Asian nations were identified as the most likely source of DENV in Guangzhou, strain and genotype alterations were frequent, and neither serotype nor genotype was dominant [39].

We established a close relationship between climate factors and the incidence of dengue fever. Specifically, we observed a lag of 1.5, 3.5, and 3 months between dengue fever and temperature, relative humidity, and precipitation, respectively. These findings confirm the widely accepted notion that temperature, precipitation, and humidity, as representative climate variables, play a crucial role in influencing the occurrence of dengue fever [1]. Many previous studies have explored the time-lag relationship between dengue fever and various climate factors, but due to the different time series and regions, there are different results [40–44]. For instance, Taiwan, which is as the same latitude as Guangzhou, had a 3 months lag relationship between dengue and temperature [41]. Another study also in Guangdong province showed that temperature, precipitation, and humidity are associated with dengue with 2, 3, 4 months lag from 1988 to 2015 [40]. Compared with these studies, we use a longer time series, which was from 1978 (the first reported outbreak in Guangdong, China) to 2018, to obtain the phase information in time–frequency by using XWT. This provided a more accurate and robust relationship between dengue and climate factors over long-time scales.

In contrast to the evident time-lag observed between temperature, humidity, precipitation, and dengue fever, the relationship between ONI and dengue fever exhibited a more intricate nature. Previous studies have also reported divergent findings regarding their association in various regions [40, 45, 46]. For instance, a study conducted in Guangdong, China from 1992 to 2011 discovered a significant coherence between ONI and dengue fever, with a lag of 12 months [40]. The reason for the difference from our study may be that seven large-scale outbreaks in Guangdong province (over 10,000 cases per year) were included in our study. These seven years which had abnormally high numbers of cases may be caused by a variety of reasons, and the EI Niño was not the only factor, for example one study conducted in Guangzhou demonstrated that the outbreak was a combination of many factors, including the improved transmission capacity of mosquitoes, increased monitoring due to the high media attention and so on [47]. In addition, multivariate ENSO Index was utilized in a research in Thailand to discover an association between ENSO and dengue with a 1–11 month lag [46].

The study revealed that precipitation, temperature, and relative humidity had high explanatory power to the incidence of dengue fever in Guangdong province. This finding aligns with a previous study conducted in Guangzhou, which demonstrated a positive association between cumulative precipitation and the number of days with light or moderate precipitation with dengue fever [48]. Increased rainfall can contribute to the proliferation of vector breeding habitats, thus influencing the incidence of dengue fever [1]. Previous studies have found a parabolic relationship between temperature and dengue fever incidence [5, 19]. Because within a certain temperature range, an increase in temperature can accelerate virus replication and shorten the external incubation period. Nevertheless, mosquito survival rates drop in extremely hot weather, which reduces the risk of transmitting dengue illness [8, 19, 49]. Prior research has also shown a parabolic pattern indicating a non-linear link between relative humidity and dengue disease. Suitable humidity can influence mosquitoes in many ways, including their life cycle, biting rate, flying distance and so on [35]. For example, one study discovered that mosquitos bite 19 times per hour in dry settings and 60 times per hour in wet conditions, demonstrating that humidity might affect dengue through modifying mosquito behavior [50].

Compared to other climate factors, the El Niño phenomenon (ENSO) exerts distinct influences in terms of intensity, duration, and time lag. Several studies have provided evidence of a positive association between the El Niño Index (ONI) and dengue fever, with increased dengue cases occurring during El Niño events in Southern Coastal Ecuador [51]. El Niño has been found as one of the main causes of dengue fever in Thailand, with ENSO events worldwide responsible for 22% of the monthly incidence variation in eight northern interior provinces [46]. It is important to note that these differences in findings may be attributed to variations in research areas and time series. The indirect influence of El Niño on mosquito behavior primarily stems from climate variations. For example, the ENSO event in Kaohsiung, Taiwan in 2005 resulted in increased humidity, which gave more ideal conditions for mosquito growth and reproduction, consequently contributing to the rise in dengue disease cases [52].

Surprisingly, our study not only examines the individual impact of climate factors but also investigates the interaction between them. We discovered that the interaction of any two variables exhibits nonlinear enhancement, with the interaction between precipitation and humidity being the most significant. Furthermore, the explanatory power of the El Niño Index (ONI) is greatly enhanced when it interacts with any climate factor. These findings lead us to comprehend three crucial aspects. Firstly, the transmission of dengue fever is influenced by both common climate factors with regular cycles and extreme climate events with irregular cycles. This finding confirms the close association between El Niño and climate change. Secondly, research should not solely focus on the relationship between individual climate factors and dengue fever but should also consider the combined influence of multiple factors. Thirdly, the water environment plays a critical role in dengue transmission, and this significant impact may be related to various hydrological factors such as soil type and vegetation. Therefore, timely monitoring of El Niño and climate change is imperative for controlling the spread of dengue fever.

The study employed a long-term series analysis to investigate the cyclical patterns of dengue fever, contributing to the body of evidence linking climate and dengue from both qualitative and quantitative perspectives. Our findings reveal notable inter-annual and intra-annual variations in dengue incidence and its susceptibility to various climate factors. Particularly, the interaction between precipitation and relative humidity emerges as the most influential factor. These findings enhance our understanding of dengue ecology and offer valuable insights for early warning and control measures. To prevent a resurgence of dengue fever amid the challenges posed by global warming, such as increased temperatures and precipitation, concerted efforts are required to bolster the public health system's capacity, raise awareness about dengue fever, encourage dengue vaccinations, and foster a healthier living environment.

This study has several limitations that should be acknowledged. As this study is based on a time series analysis, the findings may not capture the spatial variations of dengue fever at more granular levels such as prefecture-level cities, districts, and counties, due to the availability of only provincial-level dengue incidence data. Furthermore, due to data constraints and our specific aims, we did not determine the direct or indirect impact of El Niño's climate variation on dengue incidence. Future studies are expected to obtain more comprehensive data that allows for individual and combined analysis of various factors influencing dengue fever, including spatial variations and its temporal association with climate and weather. Despite these limitations, the findings of this study provide valuable insights for dengue fever warning systems and public health preparedness efforts.

## Conclusions

In the context of global climate change, the epidemic period of dengue fever has gradually lengthened over the past 40 years. The incidence of dengue is influenced by a combination of climate factors such as precipitation, temperature, relative humidity and El Niño. Therefore, future dengue prevention and control strategies should take these climate changes into account and develop corresponding measures. In addition, considering the lag relationship between the incidence of dengue fever and climatic factors, mosquito prevention and control should be carried out within a specific window period of 1.5 months in areas with high temperature, high humidity and heavy rainfall.

## Data Availability

The datasets used and analyzed during the current study are available from the corresponding author on reasonable request.

## Abbreviations

ONI: Oceanic Niño Index
ENSO: El Niño-Southern Oscillation
VIF: Variance inflation factor
COI: Cone of influence
CWT: Continuous Wavelet Transform
XWT: Cross Wavelet Transform
DENV: Dengue virus
